# Isolation, Assessments of Risk Factors, and Antimicrobial Susceptibility Test of *Klebsiella* from Gut of Bee in and around Haramaya University Bee Farm, East Hararghe, Oromia Regional State, Ethiopia

**DOI:** 10.1155/2022/9460543

**Published:** 2022-07-30

**Authors:** Abdallahi Abdurehman Damissie, Kedir Abdurahman Musa

**Affiliations:** Haramaya University, College of Veterinary Medicine, P.O. Box 138, Dire Dawa, Ethiopia

## Abstract

A cross-sectional study was employed from March 2021 to October 2021 to isolate and identify *Klebsiella* species found in the gut of honey bees collected from worker of honey bee (*Apis mellifera*) from hives in Haramaya University bee farm, Damota and Finqile's, managed under traditional and modern beekeeping apiculture. From the selected farm, a total of 60 samples of live adult honey bees were collected purposively. The live adult worker of the honey bee was individually surface-sterilized and complete alimentary canals of the worker bee were dissected and processed for *Klebsiella* isolation. Descriptive statistics were used to describe the occurrence of *Klebsiella* species and the proportion of *Klebsiella* found in the gut was analyzed for the association with study variables by the Pearson chi-square test. The overall prevalence of *Klebsiella* spp. was 50% from samples. The prevalence of *Klebsiella pneumoniae* was 26.7% and that of *Klebsiella oxytoca* was 23.3% from isolated using bacteriological examined samples. The isolates were characterized for the antimicrobial susceptibility test using the disc diffusion method. Among the isolated colonies, *Klebsiella pneumoniae* had the highest resistance to ampicillin (84.2%) and showed less resistance to gentamycin and trimethoprim sulfamethoxazole (26.3%). *Klebsiella oxytoca* was highly resistant to ampicillin (54.5%) and erythromycin (54.5%) and showed low and equal resistance to gentamycin and amoxicillin (18.2%). Molecular characterization should be conducted to identify *Klebsiella* spp. from honey bees. Monitoring antimicrobial effectiveness is recommended to tackle the existing problem in apiculture farms, and its possible public health threat should be noted for community by public health professionals.

## 1. Introduction

Honey bees are categorized under the genus *Apis*, which are social insects, that are prominent for their honey production, and nine species of honey bees are currently known such as *Apis dorsata*, *A*. *laboriosa*, *A*. *mellifera*, *A*. *florea*, *A*. *andreniformis*, *A*. *cerana*, *A*. *koschevnikovi*, *A*. *nigrocincta*, and *A*. *nuluensis* [1]. Among these, *Apis mellifera* are very important organisms that have a crucial role in reducing global food nutrition challenges and food security in the livestock industry, ecosystem service, and agriculture production [2]. Also, its indispensable products are used for nutrition and as a traditional medicine to treat diseases in humans and animals [3]. The honey bees have been great cons to enhance the yield in animal-pollinated crops which account for 35% of the global food production [4, 5]. Mass fatality cases of bees have been reported periodically in the world [6]. The bee population in eastern Africa is declined as that in other parts of continents [7]. The predisposing factors of collapsing bee families have been observed due to complex factors such as the decrease or absence of a honey plant base; the low genetic resistance of bee colonies; the impact of *Varroa jacobsoni* ticks on family resistance; the action of electromagnetic waves and radioactive radiation; pesticides and also bacterial pathogens can cause this problem [8].

Like any other animal, the gastrointestinal tract of adult honey bees is a complex ecosystem that harbors diverse microbial communities including bacteria [9].

There is a huge genetic similarity and density in the colony, and honey bees are affected by relatively few bacterial pathogens. Pathogenic bacteria such as *E*. *coli*, *Klebsiella pneumonia*, *Enterococcus faecalis*, and *Serratia mercescens* cause mortality in adult honey bees [10]. There were studies reported by researchers on bacterial diseases of bees becoming widespread in the apiaries of Ukraine and some countries in Europe and America [8, 11], leading to significant economic loss to beekeepers. In the United States, there were high annual financial losses of honey bee colonies that have averaged ∼33% annually since 2006 and have increased by approximately 12% [12, 13]. Bee diseases caused by enterobacteria were reported in 2015 in the Krasnodar territory (Russian Federation) for the first time in Eastern Europe [14]. Literature sources have reported on upper respiratory and intestinal tract disease in cattle and pigs with enterobacteriosis [15]. There are also several reports of enterobacter-induced intestinal diarrhea in humans [16]. There are not any more reports about the disease caused by bee enterobacteriosis in the published article. The microbiota of the intestine plays a crucial role in the health and nutrition of the host. Evidence has been identified of a phylogenetically grouped shift in the bacterial association of honey bees, causing a decrease in bifidobacteria and alpha-proteobacteria [17]. It has been found that the bee intestine contains a proportion of at least 10 genera of bacteria in line with the families Enterobacteriaceae, genera *Klebsiella*, *Enterobacter*, *Providencia*, *Proteus*, *Citrobacter*, *Hafnia*, *Escherichia*, *Pantoea*, *Morganella*, and *Serratia* [18]. In the meantime, the violation of quantitative and qualitative microbial contents of the intestine towards pathogenic representatives leads to the disease [11, 19]. These pathogens enter into the deeper cells of the digestive tract of bees and manifest dysbiosis. It should also be grasped that they have enhanced their difference of habitation to include hemolymph, ovaries, salivary glands, etc. [11, 20, 21]. Also, their pathogenic effect on the bee organism is manifested in acute diarrhea and general weakness of the bee colony [11]. Nevertheless, there is no single factor recognized as the cause of the high annual loss of honey bee colonies. Additionally, clinical features of the disease are described depending on the time of year and the treatment and prevention measures performed in apiaries [19].

The *Klebsiella* spp. is a non-spore-forming, facultatively anaerobic, nonmotile, Gram-negative straight rod that possesses a prominent polysaccharide capsule and its commensal pathogen found as normal flora in the gastrointestinal tract of animals and humans [22].

The widely held belief is that *K*. *pneumoniae* is a stealth pathogen, which fails to stimulate innate immune responses [23]. The predisposing factor of the bee colonies' immunological instability for several years was unproportional both in quantitative and qualitative composition of intestinal bacteria as well as by insect dysbiosis [24].

There is a paucity of information and studies on honey bee diseases that were known to be caused by bacterial pathogens in adult worker bees [25]. Some of these pathogens were riskier than others and their infections may lead to colony collapse [20]. The main cause of colony collapse disorder or decreased bee colonies, including pathogens, have been reported all over the world for several years by many researchers who have studied bee health but the problem is still disseminated all over the continents.

In Ethiopia, beekeeping is an integral part of the lifestyle of the farming communities, and except for a few extreme areas, it is a common practice in every place where humankind has settled. In addition, Ethiopia has probably the longest tradition of all the African counties in beeswax and honey marketing.

The major constraints that affect the beekeeping subsector in Ethiopia are lack of beekeeping knowledge, shortage of skills and manpower, shortage of bee equipment, pests and predators, pesticide threat, poor infrastructure development, shortage of bee forage, and lack of research extension on diseases by bacterial and other microorganisms.

Recently, the Ethiopian government is intensively working on land conservation in different parts of the country which is forming suitable conditions for beekeeping and also organizing jobless urban and landless rural youth and women to engage them in bee equipment production and beekeeping activities. A significant number of people are currently occupied in honey and beeswax collection, Tej (honey wine) making, and honey and beeswax processing and marketing. However, despite the probability of the presence of honey bee pests, disease, and other problems, the studies so far conducted on such problems in Ethiopia were very few.

Studies in Ethiopia showed that the majority of livestock owners have the habit of using antimicrobials to treat animal diseases [26, 27]. However, antimicrobial usage is characterized by shortcomings such as the inability to define the specific purposes of prescribed drug, a lack of awareness of the risks of antimicrobial resistance [26], the use of human preparation for veterinary purposes, inappropriate dosages, incomplete treatment regimens, lack of awareness of the recommended withdrawal periods, and limited access to antimicrobial varieties [26, 27].

There is a study report in Ethiopia that addresses the pathogen that affected honey bees [28]. Moreover, these studies failed to provide detailed information on the risk factors for honey bee *Klebsiella* and there is limited information on the antimicrobial susceptibility profiles of honey bee gut isolates.

To the best of researchers' knowledge, yet, there is no such study conducted in a study area as well as at the country level in general. Additionally, the data of this study were used as a preliminary study to provide baseline data for future research to explore pathogenic bacteria found in the gut of honey bees. Therefore, the objective of this study was to isolate and assess the associated risk factors and antimicrobial susceptibility test of *Klebsiella* species from the gut of honey bees in and around Haramaya University bee farm, East Hararghe, Oromia regional state, Ethiopia.

## 2. Material and Methods

### 2.1. Study Area Description

**Figure 1:**
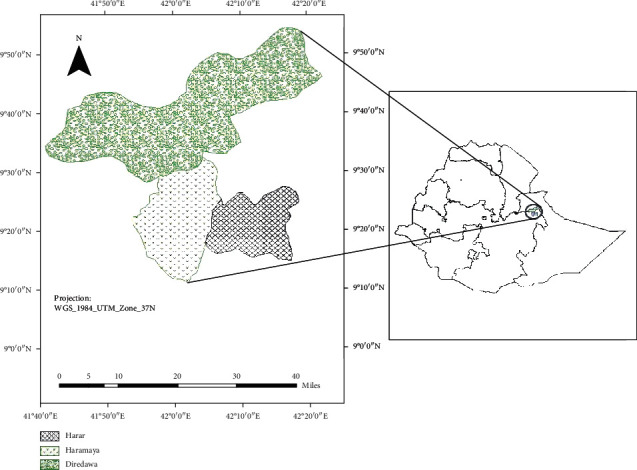
Map of the study area source [30].

Haramaya University Damota and Finkile (Figure 1) is located in the Eastern part of Hararghe Zone, Oromia regional state, Ethiopia; 509 kilometers from Addis Ababa, the capital city of Ethiopia; 17 kilometers from Harar; 40 kilometers from Dire Dawa; Haramaya university, Damota, and finkile are far apart from Haramaya town 5 km, 7 km, and 8 km, respectively. It lies between 9026′N latitude and 4203′E longitude meters above sea level. The monthly average maximum and minimum temperatures of the area are 10°C and 18°C, and the area receives an annual rainfall of 800 millimeters [29].

### 2.2. Study Population

The bee colonies were kept in both traditional and modern hives in and around Haramaya University.

### 2.3. Study Design

The cross-sectional study design was employed from March 2021 to October 2021 to isolate, identify, and conduct antimicrobial susceptibility tests of *Klebsiella pneumoniae* and *Klebsiella oxytoca* from the gut of honey bees in the study area from those managed under traditional and modern beekeeping apiculture.

### 2.4. Sampling Methods and Sample Size Determination

A purposive type of study was used to collect the sample from the apiculture digestive gut of honey bees. A total of 60 digestive gut samples of honey bees were collected from the selected site (20 Haramaya University apiculture, 20 Damota, and 20 Finqile) proportionally.

### 2.5. Sample Collection

Adult workers of healthy honey bees were collected from each site in the early morning. Samples were collected from bee colonies using sterile scissors and protective clothes and then labelled by types of hives from respective sites. The collected samples were placed in plastic boxes or sterile tubes with punched lids and placed within small pieces containing sugar cake [31]. Then, samples were transported to the Microbiology Laboratory, College of Veterinary Medicine, Haramaya University, using an ice box. If immediate inoculation of samples on media was not convenient, samples were kept at +4°C until processed for isolation.

### 2.6. Isolation and Identification of *Klebsiella* Species

The individual surface of live adult honey bees was sterilized by using 70% ethanol for 3 minutes and washing it 3 times with sterile water. The gut of honey bees was aseptically dissected by using a clipping stinger with sterile forceps and macerated with sterile scissors in 0.8% NaCl solution and kept at −4°C [31]. The samples were homogenized in 3 ml of peptone water and streaked on MacConkey agar, and incubated at 37°C for 24 hours. The isolated colony were streaked on xylose lysine deoxycholate (XLD) agar and incubated aerobically at 37°C for 24 h [32]. Then, isolated colonies of *Klebsiella* spp. were identified by means of colony morphology and conducting secondary biochemical tests such as catalase test, citrate test, H_2_S, indole test, methyl red test, oxidase test, and Voges–Proskauer test [32]. *Klebsiella pneumoniae* colonies appeared as large, shiny, and pink color on MacConkey agar, very mucoid and yellow to green on Brillant green agar, and mucoid yellow colony on XLD.

### 2.7. Antibacterial Susceptibility Test

*Klebsiella* spp. isolates were inoculated on Brain Heart Infusion Broth (Merck, Germany) at 37°C for 16 hrs. The suspension was adjusted to a turbidity equivalent to a 0.5 McFarland standard and inoculated on Mueller–Hinton agar (Oxoid, UK) using sterile cotton swabs. *Klebsiella* spp. isolates were tested for susceptibility to gentamicin (GEN 10 *μ*g), erythromycin (E 15 *μ*g), amoxicillin (AMX 30 *μ*g), ampicillin (AMP 10 *μ*g), kanamycin (KANA 30 *μ*g), and trimethoprim sulfamethoxazole (SXT 1.25/23.75 *μ*g). The zones were estimated in millimeter judge as resistance or susceptibility; accordingly, zone of inhibition was interpreted according to standards of the National Committee for Clinical Laboratory Standards [33].

### 2.8. Data Control and Analysis

The data were coded and entered into an MS Excel spreadsheet and checked for accuracy. After validation, it was transferred and processed using computer software SPSS version 20 for analysis. A descriptive statistics was used. A chi-square test was performed to assess the association of different variables with the occurrence *Klebsiella* spp. found in the gut of honey bees. A *p* value of less than 0.05 (*p* < 0.05) was considered a statistically significant association.

## 3. Results

**Figure 2:**
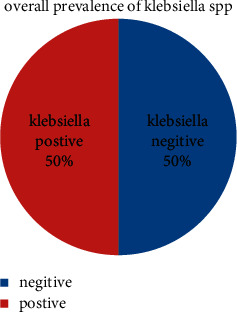
Overall prevalence of *Klebsiella* species.

A total of 60 samples of digestive tracts of honey bees (*Apis mellifera*) were collected (20 from Haramaya University, 20 from Damota, and 20 from Finkile). Two types of *Klebsiella* species were cultivated from 30 (50%) digestive tracts of positive samples only (Figure 2).

The study revealed that out of the 60 hive examined from selected study area, *Klebsiella pneumoniae* and *Klebsiella oxytoca*, 16 (26.7%) and 14 (23.3%) was detected, respectively. The prevalence of *Klebsiella pneumoniae* was higher in Haramaya University, 9 (56.2%), as compared with the other site. There were no isolate of *Klebsiella oxytoca* in Haramaya University. The prevalence of *Klebsiella* spp. were statistically significant differences (*p* < 0.05) among the studied site (Table 1). The finding showed that the number of isolate of *Klebsiella pneumoniae* was 6 (37.5) medium and 10 (62.5) in good management and for *Klebsiella oxytoca*, 4 (28.6) medium and 10 (71.4) in good management. The *χ*^2^ correlation analysis on management-related risk factors showed that there were no variations on the occurrences of *Klebsiella pneumoniae* and *Klebsiella oxytoca* among two categories. The prevalence of *Klebsiella pneumoniae* and *Klebsiella oxytoca* was not statistically significant in variation (*p* < 0.05) among hygienic practices and hive type (Table 1).

A total of 30 (16 *Klebsiella pneumoniae* and 14 *Klebsiella oxytoca)* isolates from the gut of honey bees were tested for antimicrobial susceptibility test for six different types of antimicrobials. In the present study, *Klebsiella pneumoniae* and *Klebsiella oxytoca* isolates were resistant to antibiotics. *Klebsiella pneumoniae* isolates were highest sensitive towards gentamicin (63.2%), sulfamethoxazole (47.4%), amoxicillin and kanamycin (26.3%), erythromycin (15.8%), and ampicillin (5.3%). The *Klebsiella pneumoniae* isolates were highly resistant to ampicillin (84.2%), amoxicillin (52.6%), and kanamycin (42.1%). *Klebsiella oxytoca* isolates had the highest sensitivity toward amoxicillin (72%), sulfamethoxazole and gentamycin (63.6%), ampicillin (27.3%), and kanamycin and erythromycin (18.2%). Also, *Klebsiella oxytoca* isolates were highly resistant to ampicillin (54.5%), erythromycin (54.5%), and kanamycin (45.5%).

## 4. Discussion

The current study indicated that out of the 60 examined gut of honey bee (20 from Haramaya university, 20 from Damota, and 20 from Finkile), 30 (50%) samples were positive for *Klebsiella* species (Figure 2). Among the isolates, 16 (26.7%) and 14 (23.3%) were indicated for *Klebsiella pneumoniae* and *Klebsiella oxytoca*, respectively. The occurrence of *Klebsiella pneumoniae* was higher in Haramaya University, 9 (56.2%), as compared with Damota and Finkile. These are similar to that reported in [34] where *Klebsiella pneumonia* was more abundant and prominent in the digestive guts of adult honey bees from Nigeria. The prevalence of *Klebsiella oxytoca* was 50% in both hive types (modern and traditional). The current finding was higher than that reported in [21] from Mexico which reported about 38%. The *Klebsiella pneumoniae* isolate was higher in the modern hive type (75%) than in the traditional type. The chi-square analysis revealed that the prevalence of *Klebsiella* spp. was statistically significant in differences (*p* < 0.05) among the studied area (Table 1). The finding showed that the number of isolates of *Klebsiella pneumoniae* was 6 (37.5%) medium and 10 (62.5%) in good management and for *Klebsiella oxytoca*, 4 (28.6) medium and 10 (71.4) in good management. The *χ*^2^ correlation analysis on management-related risk factors showed that there were no variations in the occurrences of *Klebsiella pneumoniae* and *Klebsiella oxytoca* among the two categories. In the present study, *Klebsiella pneumoniae* and *Klebsiella oxytoca* isolates were resistant to tested antibiotics. *Klebsiella pneumoniae* were susceptible to gentamicin (63.2%), sulphamethaxazole (47.4%), amoxicillin (26.3%), kanamycin (26.3%), erythromycin (15.8), and ampicillin (5.3%), respectively. This finding determined that the highest susceptibility of *Klebsiella pneumoniae* was indicated to gentamicin (63.2%) and the lowest susceptibility was indicated to ampicillin (5.3%). *Klebsiella pneumoniae* was high susceptibility to gentamycin which was similar to the reported that all *Enterobacteriae* strains were highly sensitive to gentamycin conducted in [35] (Table 2). *Klebsiella oxytoca* isolates showed highest sensitivity towards amoxicillin (72%), sulphamethaxazole and gentamicin (63.6%), ampicillin (27.3%), and kanamycin and erythromycin (18.2%). Also, the current finding was similar to that reported in [36] who detected the highest level of antibiotic resistance of *E*. *coli* isolated from digestive tracts of honey bees for erythromycin and low-level resistance to gentamicin. The *Klebsiella pneumoniae* isolates were highly resistant to ampicillin (84.2%), amoxicillin (52.6%), and kanamycin (42.1%). The present study revealed that ampicillin and amoxicillin was highly resistant which slightly agreed with the study conducted in [37]. The high resistance of these drugs in gram-negative bacteria might be due to the transfer of resistance genes form gram-positive bacteria of *β*-lactamase genes [38]. In other hand, *Klebsiella oxytoca* isolates were highly resistant to ampicillin (54.5%), erythromycin (54.5%), and kanamycin (45.5%).

## 5. Conclusion and Recommendation

The study isolated two *Klebsiella* species such as *Klebsiella pneumoniae* and *Klebsiella oxytoca* from the digestive tracts of honey bees. *Klebsiella* spp. was prominent in the digestive guts of adult honey bees along the study areas. The present finding revealed that the isolated organisms were tested for antimicrobial susceptibility patterns for six antibiotics such as amoxicillin, erythromycin, ampicillin, kanamycin, sulfamethoxazole, and gentamicin. The highest level of resistance was determined for ampicillin, kanamycin, and erythromycin for both species, and on the contrary, they indicated fully susceptible to gentamicin and sulphamethaxazole. In conclusion, there is a paucity of information on such studies worldwide. As well, these studies provide baseline data for comparison in future in the study area, country, and world on the prevalence of these important zoonotic pathogens in apiculture farms. Thus, based on the above conclusion, the following points are recommended:

- To address this information, this field of study needs more experiments for exact decisions.
- Such study will be conducted to investigate the source infections on isolation and identification of symbiotic or pathogenic microbiota of honey bees including molecular identification.
- Every veterinarian should conduct a variety of research regarding bee health due to economically important insects.
- The government should pay attention to bee health, especially by providing bee health as a course in different colleges and universities.

## Figures and Tables

**Table 1:** The apiculture-related risk factors for the occurrence of *Klebsiella* spp.

Variables		No. of sampled	No. of *K*. *pneumoniae*		No. of *K*. *oxytoca*		*χ* ^2^	*p* value
			Positive	% Within variables	Positive (%)	% Within variables		
Area	HU	20	9 (56.2)		0 (0)		11.1	0.026
	Damota	20	4 (25)		7 (50)			
	Finkile	20	3 (18.8)		7 (50)			

Hive type	Traditional	24	4 (25)		7 (50)		2.2	0.329
	Modern	36	12 (75)		7 (50)			

Management	Medium	24	6 (37.5)		4 (28.6)			
	Good	36	10 (62.5)	26.7	10 (71.4)	23.3	1.4	0.507

**Table 2:** Antibiotic susceptibility testing of *Klebsiella* species from the gut of bees.

Antimicrobial disk and symbol	Disc content (ug)	*Klebsiella pneumoniae*			*Klebsiella oxytoca*		
		Resistance %	Intermediate %	Sensitive %	Resistance %	Intermediate %	Sensitive %
Ampicillin	10 *μ*g	84.2	10.5	5.3	54.5	18.2	27.3
Amoxicillin	—	52.6	21.1	26.3	18.2	9.1	72.7
Trimethoprim sulfamethoxazole	23.75 *μ*g	26.3	26.3	47.4	27.3	9.1	63.6
Gentamycin	10 *μ*g	26.3	10.5	63.2	18.2	18.2	63.6
Erythromycin	15 *μ*g	31.6	52.6	15.8	54.5	27.3	18.2
Kanamycin	30 *μ*g	42.1	31.6	26.3	45.5	36.3	18.2

## Data Availability

The data are included in the tables within the manuscript.
